# Hints to Junior House Surgeons

**Published:** 1907-10-26

**Authors:** 


### Resident Medical Officers' Department.

[The Editor accepts no responsibility for any opinions expressed in these columns, which are freely open to all resident medical officers. Discussion and contributions are invited, and, if the latter are accepted, they will be paid for.]

### HINTS TO JUNIOR HOUSE SURGEONS.

BY A SENIOR.

I had the good fortune to have more than a year's experience of private practice before entering on hospital work. This taught me wherein I might profit most by my stay in hospital. Most men take residency work in order to qualify themselves for general practice. Of these some pick up involuntarily whatever happens to come their way. Others, again, have preconceived notions, often faulty, as to what is or is not best for them to devote attention to. For these a few lessons from my experience may be of use.

I notice that laboratory work has a great fascination for junior house surgeons and physicians. They rush their ward work in their haste to prepare slides, examine stomach contents, and make blood counts, etc. Now, far be it from me to throw cold water on this, but I think there is a tendency to overdo it. In practice, if the practice is worth having at all, there is no time for carrying out such examinations, and indeed, even if there were, the results, especially of blood counts, are often quite unsatisfactory. The only man who can do reliable work at blood counting is the man who does it every day. Therefore in practice it is far better to send all such clinical pathology work to the Clinical Research Association or some similar organisation where the work will be done thoroughly and surely. So true is this that there is growing up a race of specialists whose sole work lies in reporting on secretions, discharges, and other pathological specimens; and municipal authorities recognise this when they authorise their medical officers of health and the staffs of their isolation hospitals to report on throat swabs free of charge. Much of this work the resident does at the expense of his ward work, and to the neglect of his patients.

In my opinion the greatest thing the resident can learn in hospital is how to deal with patients. One must remember that they are drawn from that class which provides the greater part of the work of most practices. He must get to know these people, earn their confidence, acquire what patients call "manner," which is the standard by which they will judge him. In other words, no amount of knowledge will make up for lack of skill in handling men, and the house surgeon has peculiar opportunities for acquiring this skill. In medicine art often succeeds alone, science never.

In the smaller hospitals attention to the dispensary falls among the resident's duties. To many this is, quite mistakenly, a bore. Most practices, in England at least, are dispensing practices where a knowledge of the price of drugs, of the most suitable medicines to stock, and a certain speed in their dispensing are invaluable. How many house surgeons know, for example, that quinine is expensive, that tr. opii is rising in price, and that it is more economical to buy liq. strych. hydrochlor. than tr. nucis vom.? Yet knowledge of this sort may mean all the difference between success and failure, financially.

Another thing that will repay attention is *x*-ray work. Every year small hospitals are being reared all over the country, and most of them are ambitious enough to have an *x*-ray apparatus. Here lies a great chance for the junior man. The older practitioner has possibly never seen the apparatus; he may even be chary of its use and doubtful of its value. Let the junior man be the man in his district who can manipulate the rays. It will mean much kudos, and money, too, when his chance comes later on.

As regards the type of case he should endeavour to see most of, I have no hesitation in urging him to devote his attention to simple ailments: and these he will see in the out-patient department. General practice consists in the treatment of simple ailments. Whilst he is in hospital it is well a man should see something of intracranial and orthopædic surgery, of prostatectomies and excisions of kidneys in surgery, and of the more obscure organic medical diseases, but in private the common cold, quinsy, and constipation will be his portion, and the out-patient department will furnish him with abundant practice in these. So many men treat these conditions in a rule of thumb fashion, which never breeds success. The man who can choose with fine discrimination his drug and its dose and can exhibit it without discomfort is the man who makes his mark in private work. The surgical subjects which especially merit the attention of the future general practitioner are fractures, and the operative treatment of hæmorrhoids, of enlarged tonsils, and of strangulated hernia, while casualty work of every kind is a valuable experience.

